# Supplementary material for: The Global, Regional, and National Uterine Cancer Burden Attributable to High BMI from 1990 to 2019: A Systematic Analysis of the Global Burden of Disease Study 2019
Source: J Clin Med. 2023 Feb 27;12(5):1874. doi: 10.3390/jcm12051874 (PMC10003834; doi:10.3390/jcm12051874)
Supplement: Supplementary file 1 [file jcm-12-01874-s001.zip › Figure S1.pdf]

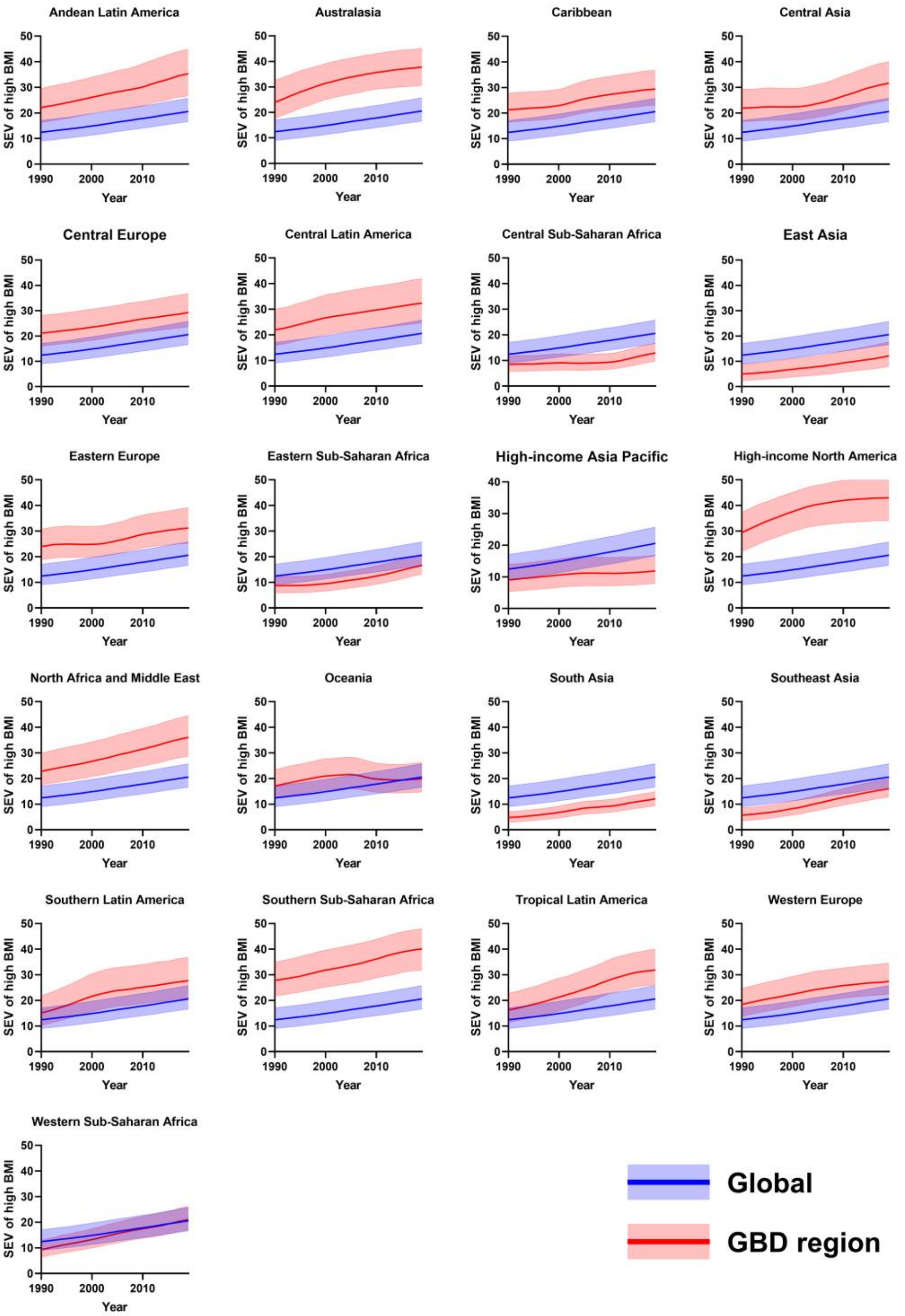

Supplementary Figure S1. SEV of high BMI for women (per 100) in GBD regions from 1990 to 2019. Red represents GBD regions and blue represents global levels. SEV, summary exposure values.
